# Blockchain-based trusted traceability and sustainability certification of leather products

**DOI:** 10.1371/journal.pone.0333192

**Published:** 2025-09-25

**Authors:** Ruba Islayem, Haya R. Hasan, Ahmad Musamih, Khaled Salah, Raja Jayaraman

**Affiliations:** 1 Center for Digital Supply Chain and Operations Management, Khalifa University, Abu Dhabi, United Arab Emirates; 2 Department of Computer and Information Engineering, Khalifa University, Abu Dhabi, United Arab Emirates; 3 Department of Management Science and Engineering, Khalifa University, Abu Dhabi, United Arab Emirates; 4 Department of Industrial Engineering, New Mexico State University, Las Cruces, New Mexico, United States of America; Symbiosis International (Deemed University), INDIA

## Abstract

The leather supply chain comprises numerous organizations and stakeholders, particularly when sustainability aspects are taken into account, making it a complex system. The complexity inherent in such systems can lead to inaccurate information, lack of transparency, and limited data provenance. Moreover, there has been a surge in the call for sustainable practices within leather production, propelled by growing environmental consciousness and ethical considerations. In this paper, we address these challenges by proposing a blockchain-based solution designed to ensure trusted and secure traceability and sustainability throughout the entire life cycle of leather products. By harnessing the inherent capabilities of Ethereum smart contracts and blockchain technology, such as decentralization, immutability, data integrity, and transparency, we guarantee the secure and reliable tracing of materials from the farm to the final consumer. Moreover, we provide proof of sustainability by which certification agencies monitor, audit, and approve the sustainable processes and practices carried out by the different stakeholders at all stages of production to ensure compliance with industry standards and regulations. The paper presents the blockchain-based system architecture, implementation, and validation of algorithms and smart contracts. It also evaluates the security measures and cost-effectiveness of the system to offer valuable insights into its robustness and efficiency. We have made the developed smart contracts code publicly available on GitHub.

## 1 Introduction

The leather industry plays a significant role in global manufacturing by producing essential goods like footwear, bags, and garments that are widely traded and consumed worldwide. Particularly in developing countries reliant on agricultural economies, leather processing has emerged as a crucial economic activity. The global annual leather production is estimated at 23 billion square feet [1], with much of this production concentrated in developing nations due to the labor-intensive nature of the processes involved [2]. The fashion sector, characterized by its diverse array of activities, encompasses various value chains, including the production and distribution of textiles, footwear, and the tanning of hides and skins [3]. The leather industry holds a prominent position in the global economy, with an estimated annual global trade value of approximately $350.77 billion [4]. Demand consistently surpasses supply growth in this sector.

In recent years, a noticeable shift in public consciousness towards environmental protection and sustainable development has prompted increased scrutiny of industries that adversely impact the environment. Among these industries, the global leather industry has come under heightened scrutiny due to its significant environmental footprint. The fashion industry, of which leather goods production is a significant component, ranks as the second most polluting industry after the oil sector [3]. Traditional leather production processes, characterized by resource-intensive practices such as excessive water usage and chemical treatments, contribute to environmental degradation. Specifically, the tanning process, known for its heavy use of chemicals and water, generates both liquid and solid wastes, resulting in high wastewater emissions [3]. Annually, the industry consumes a staggering 93 billion $m^{3}$ of water, employs over 8000 chemicals in raw material treatment, and generates approximately 0.80 kg of waste per kg of raw hides/skins [3,5]. Despite growing awareness of sustainability issues, research indicates that tanneries have been slow to adopt sustainable practices [3]. The industry’s hesitance to adapt is rooted in financial limitations and the entrenched resistance within its conservative culture. Consequently, the industry faces significant challenges in resource management, environmental impact, and ethical sourcing. Addressing these challenges demands precise and comprehensive solutions aligned with sustainability goals.

**Fig 1:**
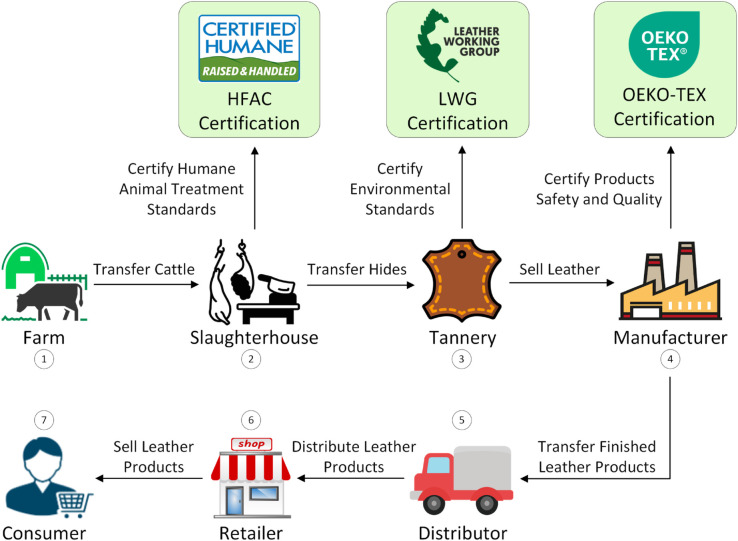
A typical flow of the leather production process with certifications.

A typical process flow for the production of leather products, including sustainability certifications, is illustrated in Fig 1. The supply chain typically starts from the farming process, where cattle are raised to produce high-quality hides used in leather production. This stage involves ensuring proper care, nutrition, and ethical treatment of the animals to yield hides suitable for leather production [6]. Subsequently, the cattle are transferred to slaughterhouses where they are humanely slaughtered, and their skins are salted and dried to prevent degradation [6]. Slaughterhouses should obtain certification from an animal welfare organization, such as Humane Farm Animal Care (HFAC) [7], to ensure humane treatment throughout farming and slaughter processes. Once the hides are obtained, they are sent to tanneries where they undergo a series of chemical treatments to transform animal hides into stable and durable high-quality leather, which is protected against water, heat, and microorganisms [8]. Due to the use of various chemicals in this process [8], tanneries should obtain certification from the Leather Working Group (LWG) [9] for adhering to environmental stewardship standards that ensure chemical safety, safe wastewater disposal, and efficient energy usage. Once leather is ready, it will be utilized by manufacturers to create various leather products such as shoes, bags, and garments. The OEKO-TEX Certification [10] held by manufacturers provides consumers with confidence in the safety and quality of the leather products they purchase. Following production, the finished leather products will be distributed to retailers, where they are showcased and made accessible to end consumers.

The leather supply chain comprises an intricate network involving various stakeholders and certification agencies, each presenting unique challenges related to ethical practices, environmental sustainability, and traceability. A traceability system within the leather supply chain is an effective solution that tracks the movement of materials from the farm to the end consumer. Enhancing transparency through implementing supply chain mapping and traceability systems results in reduced environmental, social, and quality risks, and fosters credible communication with stakeholders and consumers. This also compels stakeholders to adhere to sustainable and ethical standards by obtaining certifications from sustainability certification agencies, which should be integral parts of the supply chain. This approach enables brands to source from stakeholders meeting their criteria for animal welfare, deforestation, and other sustainability metrics which aligns with both their internal objectives and customer expectations.

Utilizing advanced technology such as blockchain is the key to enhancing transparency in the leather supply chain. Blockchain technology is a widely utilized tool for ensuring traceability and transparency in supply chains. Functioning as a distributed decentralized ledger, blockchain facilitates secure and transparent transactions without relying on centralized intermediaries. By linking blocks using cryptography and hashes, blockchain creates an immutable chain of records, ensuring tamper-proof and highly secure data storage [11]. Its intrinsic features, including decentralization, traceability, auditability, and transparency, make it a valuable asset across various industries. Additionally, the integration with the Ethereum blockchain, complemented by programmable logic via smart contracts, enables stakeholders to conduct operations transparently and with accountability. Ethereum programmable nature empowers it to govern business logic, enforce workflows, and execute agreed-upon processes among supply chain participants [12]. Smart contracts serve as protocols for digitally verifying and executing credible transactions to ensure traceability and irreversibility without third-party intervention [13].

In this paper, we propose a blockchain-based solution to ensure trusted and secure traceability and sustainability across all production stages of leather products. By securely linking details about raw materials, production processes, and labor practices to the blockchain, stakeholders gain comprehensive visibility over the entire supply chain. Ethereum smart contracts automate traceability criteria which guarantees material authenticity and facilitates secure transactions. They also aid in obtaining and validating necessary sustainability certifications for stakeholders to ensure compliance with sustainability standards and regulations. By adopting this approach, our solution establishes a robust foundation for traceability, transparency, and sustainable practices in leather production.

The primary contributions of this paper are summarized as follows:

- We propose a blockchain-based solution to manage operations within the leather products supply chain to ensure security, traceability, and immutability. Also, We provide a proof of sustainability by incorporating multiple certification agencies that oversee and periodically assess workplace activities.
- We develop Ethereum smart contracts to facilitate and automate transactions and payments among stakeholders while ensuring trusted and secure traceability and sustainability.
- We conduct security and cost analyses to assess the performance of our solution in real-world scenarios.
- We compare our solution with existing solutions, and we discuss how our solution can be extended and generalized to other applications with similar requirements.

The remaining sections of this paper are organized as follows. Sect 2 presents an overview of related work in the literature. Sect 3 provides an explanation of the design and architecture of our proposed blockchain-based solution. In Sect 4, we present the implementation details and algorithms employed within the smart contracts. Subsequently, Sect 5 outlines the testing and validation procedures carried out on the developed system. Sect 6 presents cost and security analyses, along with a comparison with the existing solutions. Finally, we conclude the paper in Sect 7.

## 2 Related work

In this section, we offer an overview of existing solutions in the literature. We review and summarize blockchain and non-blockchain-based solutions that address traceability and sustainability challenges within the leather supply chain.

### 2.1 Non-blockchain-based solutions

To address sustainability concerns within the leather industry, traditional methodologies have long relied on established recycling approaches to minimize environmental impact. These methods have focused primarily on optimizing resource utilization and waste reduction. As highlighted by [14–16], textile recycling shows potential in fostering the circular economy by reducing reliance on new materials, consequently conserving water, energy, and chemicals. However, with increasing demands for transparency, traceability, and environmental accountability, new approaches and technologies have emerged to address these evolving challenges.

Denuwara et al. [17] discussed the sustainability advantages of implementing RFID technology in the apparel industry. They mentioned how it can be used to enhance inventory accuracy, minimize stock inaccuracies, and reduce the need for safety stock, thereby decreasing waste and greenhouse gas emissions. They can also be used to extend the lifespan of clothing by providing consumers with detailed care instructions and recycling information, thus reducing carbon emissions associated with fast fashion and waste generation.

Additionally, Marconi et al. [18] proposed a structured approach to expand upon the traditional traceability concept, integrating environmental impact assessment throughout the supply chain (SC). The method comprises four steps: SC modeling, continuous data collecting and sharing using IoT devices, data elaboration for sustainability assessment, and result interpretation for optimization. The approach enhances sustainability by including energy and resource consumption data, which are crucial for detailed environmental evaluations. It aids in identifying potential weaknesses and issues to successively set corrective actions, while also informing stakeholders and consumers about the origins of raw materials.

Similarly, Papetti et al. [19] presented a web-based platform for tracing suppliers and processes throughout the SC, aiming to improve overall environmental sustainability. The platform requires quantitative data on material flows, energy consumption, water usage, waste, and emissions. It comprises two software modules, Trace and Life Cycle Assessment (LCA), and communicates with databases for SC assessment. The Trace module manages traceability data and communicates with traceability stations within each company, while the LCA module calculates the environmental impact of the SC. Through data analysis, the platform helps identify critical areas, optimize logistics, and promote sustainable procurement practices.

Regarding traceability and transparency, Thakur et al. [20] introduced a traceability system for hides aimed at providing feedback to producers about farm handling practices. Various data capture technologies were tested, including RFID, dot peening, and laser engraving. Results indicated that RFID tags enabled traceability from the farm to the hide processor. However, these tags were not suitable for the tanning process, as they were lost after processing. Similarly, hides marked with dot peening experienced readability issues after tanning. Lastly, laser engraving was effective for traceability throughout the supply chain, including the tanning process. Therefore, it was suggested to be used for traceability, especially considering its coverage of the tanning process.

The non-blockchain-based solutions discussed exhibit notable drawbacks, particularly in terms of security and trustworthiness. These approaches rely on centralized systems and databases, which are susceptible to security breaches and data manipulation. As a result, there are concerns regarding the integrity and reliability of the information stored within these systems, which can undermine trust among stakeholders. Additionally, technologies like RFID tags can introduce additional costs and time-consuming processes due to their item-centric nature, requiring processing for each individual product at various stages. Therefore, the inherent limitations of these solutions underscore the need for more secure and trustworthy alternatives, such as blockchain technology, to ensure the integrity and transparency of supply chain data.

### 2.2 Blockchain-based solutions

Research across various sectors, including fashion, luxury goods, and footwear, has explored the potential of blockchain technology to enhance supply chain traceability and sustainability. Studies conducted by Cui et al. [21] and Boissieu et al. [22] explored the application of blockchain technology in improving traceability and monitoring within industrial processes, such as the footwear supply chain and the luxury industry. They addressed crucial questions regarding blockchain adoption, its potential benefits for companies in the footwear sector, and the challenges associated with implementing blockchain solutions.

Hader et al. [23] presented a new framework leveraging blockchain technology to enhance traceability in the textile supply chain, fostering transparency and information sharing among stakeholders. This framework empowers consumers to make informed purchasing decisions while strengthening stakeholders’ relationships, improving efficiency, and mitigating risks like product recalls and counterfeit goods. Additionally, to overcome blockchain scalability challenges, the authors proposed an innovative integration of blockchain with Big Data technology to address these issues and ensure the scalability of decentralized systems at scale.

The blockchain-based STVgoDigital platform proposed by Alves et al. [24] aims to enhance traceability and sustainability in the textile industry. Its core objective is to generate a sustainability index for textile products by collecting environmental and social indicators for each product. This index provides consumers with critical insights for making informed purchasing decisions. Furthermore, leveraging Hyperledger Fabric, the solution offers traceability features enabling the tracking of the environmental and social indicators throughout the supply chain.

Additionally, Shou et al. [25] investigated the integration of LCA and blockchain to enhance traceability and assess environmental impact in the fashion sector. The authors evaluated the potential environmental benefits of circular strategies and proposed a protocol for integrating LCA and blockchain. The findings suggested significant environmental advantages from circular practices and highlighted the role of blockchain in enhancing traceability and data sharing. While the authors focused on understanding the strengths and weaknesses of LCA for identifying environmental hotspots and circular fashion strategies, no concrete implementation of blockchain integration was provided.

Conversely, Perez et al. [26] proposed a blockchain-based solution for the clothing industry to enhance traceability, reliability, and authenticity. Their approach involved utilizing a distributed ledger to securely record each step in the textile process as separate blocks in the chain. For instance, initial blocks recorded supplier details and raw material origins, while subsequent blocks encapsulated information on fabric treatments like dyeing, stamping, finishing, and coating. However, a drawback of this method is the inherent complexity associated with retrieving specific information through blocks and hashes, posing limitations on its practicality.

AL-Issa et al. [27] discussed how blockchain technology can be used to boost luxury sustainability by enabling the tracking of product history and production processes from raw material extraction to the delivery of products. This involves monitoring the inclusion of materials in finished products and the sourcing conditions of raw materials. However, the authors did not address the crucial challenge of validating sustainable practices and preventing violations during production processes. Furthermore, the lack of implementation details limits its practical applicability.

This gap underscores the necessity of our proposed solution, which involves partnering with sustainability certification agencies. This collaboration aims to furnish reliable proof of sustainability for leather products, thereby guaranteeing ethical practices throughout every stage of production. Overall, existing studies have been largely theoretical, lacking practical implementation and a specific focus on leather products. This emphasizes the necessity of a solution specifically tailored to address the challenges of the leather industry, emphasizing practical implementation and a steadfast commitment to sustainability.

## 3 Proposed blockchain-based solution

In this section, we provide a detailed explanation of our proposed blockchain solution for tracing and tracking leather products. Our solution leverages Ethereum smart contracts and utilizes immutable logs and trusted events to facilitate tracing and tracking from the inception at the farms to the ultimate consumer while ensuring compliance with sustainability practices.

### 3.1 System architecture and participating entities

**Fig 2:**
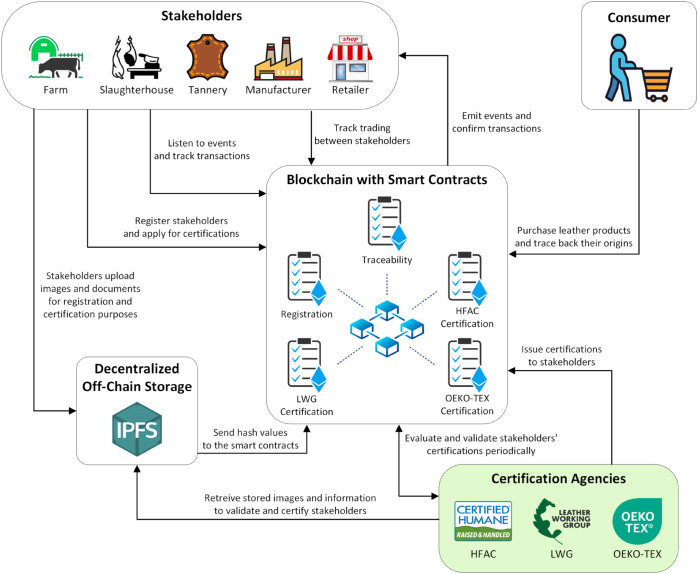
A high-level architecture for the proposed blockchain-based solution.

Fig 2 depicts a high-level architecture of our proposed blockchain-based solution that shows the relationship between the participating entities and the smart contracts. These participating entities include stakeholders and certification agencies that engage with each other through smart contracts, and possess unique Ethereum addresses to facilitate interactions within the blockchain network. Our solution also deploys five smart contracts, each equipped with unique functions executable only by pre-authorized actors. To optimize smart contracts performance and reduce costs, large-sized content such as images and documents are stored off-chain on the InterPlanetary File System (IPFS). Furthermore, our system eliminates the need for centralized servers and third-party intermediaries, as all participants interact directly with on-chain resources to access information such as logs, events, transactions, and IPFS hashes. This approach not only mitigates the limitations of traditional centralized systems but also enhances trust, transparency, and integrity. Further details on the system components are presented below.

**Stakeholders:** Farms, slaughterhouses, tanneries, manufacturers, and retailers serve as participants in the smart contracts within the supply chain. Each stakeholder has a specific role which grants them access rights to restricted functions within the smart contracts. They can interact with each other through the traceability smart contract to exchange materials, and they are provided access to on-chain information to allow them to view logs, transactions, and events. Furthermore, stakeholders engage with the registration manager and certification agencies through smart contracts to initiate registration or certification requests. For these processes, they utilize the decentralized IPFS storage system to upload necessary documents and images for verification, off-chain identification and validation purposes.

**Certification agencies:** Slaughterhouses, tanneries, and manufacturers are required to obtain certificates for their products to ensure compliance with sustainability standards and regulations. Certification requests are submitted by stakeholders to certification agencies through certification smart contracts. These certification agencies are authorized to access and review images and documents uploaded on the IPFS by stakeholders. This enables them to examine and validate the documents, which facilitates their decision-making process for either accepting or denying certification requests. Additionally, they will conduct periodic announced or unannounced inspection visits to the stakeholders to frequently validate the obtained certifications and they can revoke certifications if any violations are detected.

**Ethereum smart contracts:** Smart contracts are utilized to oversee the entire supply chain and enforce the agreed-upon rules among stakeholders. They serve as a central and essential component for tracking transaction history and managing hashes from the decentralized storage server to allow participants to access supply chain information. Our system architecture consists of five smart contracts: Registration SC, Traceability SC, and separate contracts for each of the three certification agencies - HFAC, LWG, and OEKO-TEX. The Registration SC is responsible for registering stakeholders in the system by assigning a unique Ethereum address to each one to allow them to execute specific functions within the smart contracts based on their role. This ensures restricted access to certain functions to prevent unauthorized actions on behalf of stakeholders. The Certification SCs manage certification requests, examine uploaded IPFS hashes by stakeholders, and approve, deny, or revoke certifications. Certified stakeholders are recorded on the blockchain and granted access to specific functions. Lastly, the Traceability SC manages and traces the processes of producing and exchanging materials between stakeholders to restrict transactions to registered and certified stakeholders to ensure authenticity. It traces the origin of materials by mapping each material to the previous materials used to generate it and the stakeholders it came from. Through event triggering and blockchain logging, traceability and proof of sustainability are achieved, which enable end-users to trace and verify the origin and certification status of leather products.

**On-chain storage:** All logs, transactions, and events generated by smart contracts are permanently stored on the ledger. This means that they cannot be altered or removed, which provides a secure and tamper-proof record of all activities within the system. Additionally, the on-chain storage is utilized to register participants and record any violations that occur during the production processes. This feature ensures traceability, accountability, and sustainability in the proposed solution.

**Decentralized off-chain storage system:** IPFS is an essential component of our proposed solution, providing low-cost off-chain storage while ensuring the reliability, accessibility, and integrity of stored data [28]. Within our system, stakeholders are required to store large files such as pictures and videos of their facilities and production processes, registration and certification application forms, and other detailed documents and records. Therefore, utilizing a decentralized off-chain storage system is crucial to overcome the storage constraints of the blockchain. The integrity of data is maintained by generating a unique hash for each uploaded file on the IPFS server [28]. These hashes are then stored on the blockchain and accessed through the smart contracts, and any changes made to the uploaded files are reflected in the associated hash.

**Consumer:** The consumer serves as the final stakeholder in the lifecycle of leather products. Upon purchasing leather products, consumers have the ability to track all associated records, including information about the stakeholders they originated from, the specific raw materials used, and the status of the certifications obtained by these stakeholders. This tracking can be facilitated through the unique Ethereum address of the product, which provides access to comprehensive records and details. By providing consumers with transparency and access to such detailed information, our blockchain-based system ensures trust, accountability, and proof of sustainability within the leather industry.

### 3.2 Interactions among the participating entities

This subsection presents three sequence diagrams that illustrate the primary interactions among participants and smart contracts within our proposed solution. Each participant is assigned an Ethereum address and participates in the system by invoking functions within the smart contracts. The process is divided into three main phases, detailed below.

**Fig 3:**
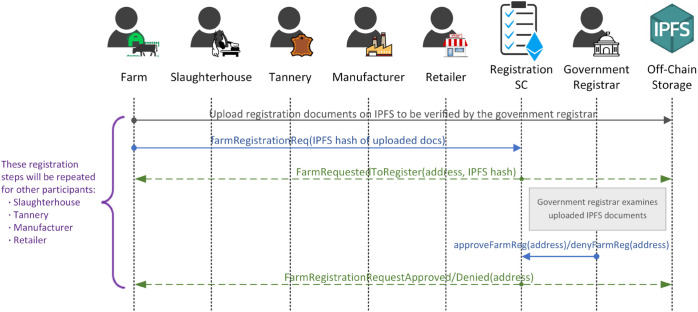
Sequence diagram illustrating the interactions between the participating entities and the registration smart contract.

**Registration phase:** The registration phase, illustrated in Fig 3, marks the initial step, where stakeholders are registered in the system. This sequence diagram illustrates the interactions among stakeholders, IPFS, the government registrar, and the registration smart contract in the scenario of farm registration. The smart contract, owned by a registrar authorized by the government, facilitates stakeholder registration. Initially, an offline agreement between the farm and the registrar outlines the registration steps and the terms and conditions. Subsequently, the farm submits the agreed-upon registration documents, possibly including images and videos, to IPFS and receives an IPFS hash for the uploaded documents. Then, the farm initiates the registration process by calling the *farmRegistrationReq()* function to provide the IPFS hash of the registration documents. This function triggers the *FarmRequestedToRegister* event to broadcast the availability of registration requests and providing relevant parameters. The registrar receives the notification and accesses the uploaded documents using the IPFS hash, examines them, and decides whether to approve or reject the registration request. If the documents meet the requirements, the registrar calls the *approveFarmReg()* function, registering the farm on the system and storing its address on the ledger for authorization purposes. Conversely, if the requirements are not met, the registrar can deny the registration by calling the *denyFarmReg()* function. Finally, the *FarmRegistrationRequestApproved* or *FarmRegistrationRequestDenied* event is triggered to inform all participants of the registrar’s decision. These steps are repeated for the registration of all other stakeholders. Once stakeholders are registered, they gain access to pre-authorized functions in the subsequent phases.

**Fig 4:**
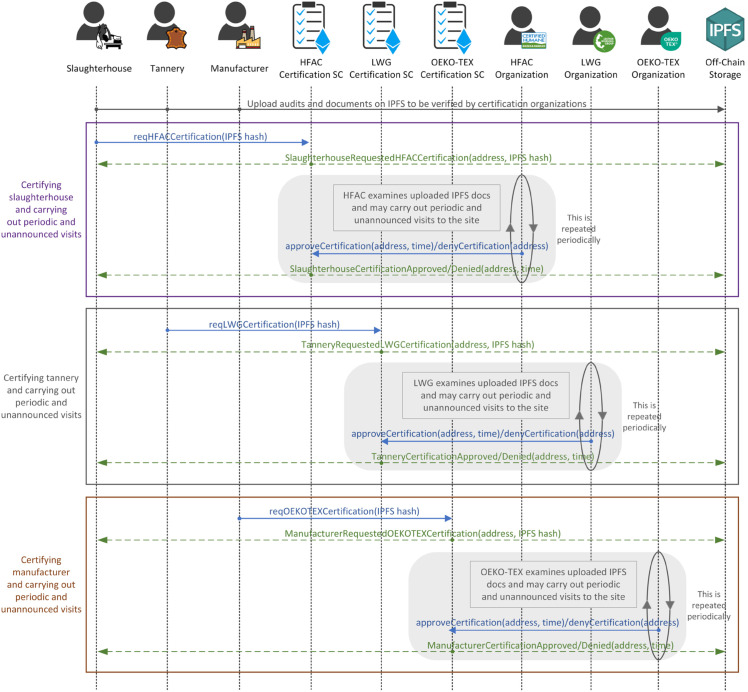
Sequence diagram illustrating the interactions among the participating entities and the certification smart contracts.

**Certification phase:** Fig 4 outlines the process of stakeholders obtaining certifications from certification organizations through interactions with certifications smart contracts. Each certification organization owns and deploys the respective smart contract. Before initiating the certification process, stakeholders familiarize themselves with the requirements and terms of the certifications off-chain. Subsequently, stakeholders upload application forms and required records to IPFS. Then, the on-chain process initiates as the slaughterhouse calls the *reqHFACCertification()* function to provide the IPFS hash value. This triggers an event to notify the certification organization of available certification requests. Following this, an off-chain process takes place where the certification organization examines the IPFS documents and conducts on-site inspections. Upon reaching a decision, the certification organization calls the *approveCertification()* or *denyCertification()* functions to trigger events that notify all participants. Additionally, certification organizations have the authority to request documents and audits from certified stakeholders at any time, and stakeholders are required to upload these documents to the IPFS and provide the corresponding hash.

To ensure a trusted proof of sustainability, periodic inspection visits to the sites will be conducted, with the possibility of unannounced visits at any time to ensure compliance with sustainability and environmental standards. If any violations are detected, the certification organizations reserve the right to revoke certifications immediately. Moreover, each certification includes a validation period, indicated by the triggered event, which necessitates stakeholders to reapply for certifications once they expire. To enforce this mechanism, smart contracts validate the certification’s expiration before any production or exchange operation by checking the current blockchain timestamp against the stored expiry date. If a stakeholder attempts to transact with an expired certification, the function is blocked, and an error is returned to prevent unauthorized activities. Once a certification expires, the stakeholder enters a short grace period during which all functions remain disabled. Within this period, a renewal can be requested without requiring a full reinspection. However, if the grace period ends without renewal, the certification is revoked, and the stakeholder must reapply from the beginning. This approach ensures timely renewal, prevents misuse of expired certifications, and maintains continuous compliance with sustainability standards.

The inspection visits and validation period policies are essential for ensuring that stakeholders remain monitored throughout all stages of the production process and for preventing violations. Furthermore, certified stakeholders will be recorded in the blockchain using mappings, and only certified stakeholders will be authorized to participate in the production and material exchange phase. This authorization mechanism provides assurance to end consumers and other stakeholders that all products originated from trusted and verified sources. Consumers can also utilize the mappings to verify the status of certifications, which enhances transparency and trustworthiness throughout the supply chain.

While the current model assigns each certification authority to a single organization, the system is designed to be compatible with future extensions supporting multi-agency certification. Each certification smart contract can be modified to record approvals from multiple independent agencies and issue the final certification only after reaching a predefined agreement threshold. This structure allows for increased decentralization, reduced reliance on individual certification agencies, and enhanced trust in the certification process. Moreover, it is important to note that the system currently depends on off-chain inspection results being manually submitted by certification agencies. This introduces a potential vulnerability known as the "oracle problem", which is the gap between physical reality and digital representation on the blockchain. Although certification actions are publicly logged to ensure transparency, the system assumes the honesty of certification agencies’ input. In future versions, this limitation should be addressed by incorporating trusted hardware, decentralized oracle networks, or cryptographic attestations from inspection devices to provide stronger assurance that off-chain inspections and sustainability metrics are accurately reflected on-chain.

**Fig 5:**
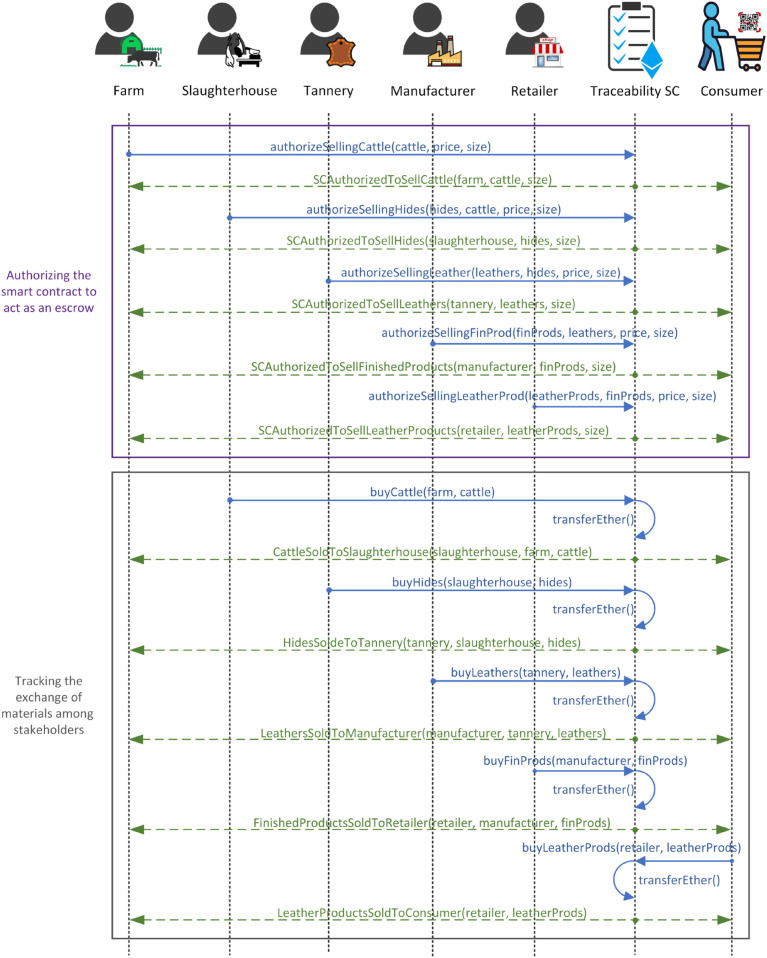
Sequence diagram illustrating interactions among participants and the traceability SC for exchanging materials during the production phase.

**Production phase:** Fig 5 illustrates interactions among stakeholders, the traceability smart contract, and the end consumer. In this phase, the smart contract acts as an escrow, overseeing and tracking the production and exchange of materials between stakeholders and the end consumer. Each raw material lot is assigned a unique Ethereum address for tracking purposes.

To begin the process, stakeholders must first authorize the smart contract to sell their products. Once authorized, other stakeholders can directly purchase products from the smart contract without interacting with the sellers. For example, a tannery would execute the *authorizeSellingLeather()* function, which specifies attributes such as the address of the leather lot to be sold, the total price, the lot size (single or multiple leathers), and the address of the previous material lot used in production. Upon execution, the *SCAuthorizedToSellLeather* event is triggered to notify stakeholders of available leather lots for purchase. A mapping is created for each material, containing all relevant details of the products. Information like the address of the previous raw material lot is crucial for validation to allow consumers to trace product origins using the mappings and unique addresses.

Once the smart contract is authorized to sell the leather, manufacturers can execute the *buyLeather()* function by specifying attributes such as the address of the leather lot to purchase and the tannery address. The smart contract validates these addresses and transfers ether to the farm address if all conditions are met. After a successful transaction, the *LeatherSoldToManufacturer* event is triggered to notify relevant stakeholders, and the manufacturer’s address is added to the leather mapping for tracking and validation purposes. Similar procedures are applied to other authorization and purchasing functions which enable facilitating the exchange of other materials. These processes occur iteratively, with stakeholders executing these functions as needed. All functions of the Traceability smart contract are restricted to registered and certified stakeholders. Through these interactions and restrictions, consumers and stakeholders ensure the trusted authenticity, traceability, and proof of sustainability of raw materials and final products. They utilize mappings, logs, and Ethereum addresses for verification, which ensures transparency throughout the supply chain.

## 4 Implementation details

In this section, we introduce and discuss the algorithms developed to implement our proposed solution, along with their implementation and coding details. These algorithms define the working principles and form the foundation for the development of Ethereum smart contracts. The smart contracts are written in Solidity language and compiled and tested using the Remix IDE [29]. Remix is an online web-based development environment that enables users to write and execute smart contract code, as well as provide debugging and testing capabilities for Solidity code. We present below detailed algorithms representing various functions and working principles of the smart contracts. Our focus here is on scenarios specific to tanneries, however, similar functions are employed for all other stakeholders. The implemented smart contracts code is made publicly available on GitHub (https://github.com/rubak3/LeatherTraceability).

**Submitting registration requests:** Algorithm 1 represents the initial step of the registration phase, which enables stakeholders to request registration on the system to gain permission for subsequent interactions. This function can be executed by any stakeholder, which must provide the IPFS hash of the registration documents as input. The function verifies if the tannery is not already registered by checking the mapping of registered tanneries. If the tannery is not registered, the function emits an event containing the tannery address and the associated IPFS hash.

**Algorithm 1. Submitting registration request.**



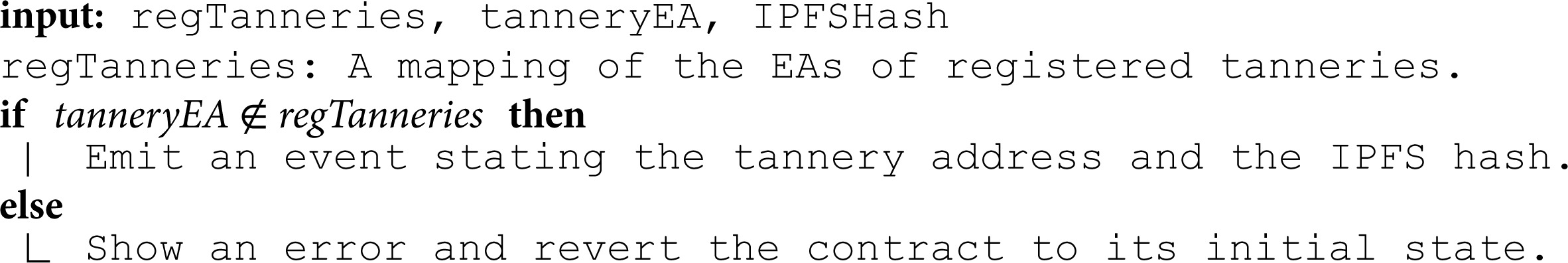



**Approving registration requests:** Algorithm 2 describes the function for approving registration requests. This function is restricted to the government registrar, who serves as the owner of the smart contract. If the registration is approved, the function is executed by taking the tannery address as an input. The tannery address will be added to the mapping of registered tanneries by setting its value to true, and an event will be emitted confirming that. Being added to the mapping authorizes the tanneries to execute other functions in the other smart contracts. These functions can check the mapping to validate authorization before proceeding.

**Algorithm 2. Approving registration request.**



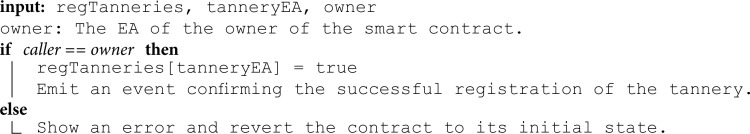



**Submitting certification requests:** Algorithm 3 shows the function used to request LWG certification, which is restricted to registered tanneries only. Tanneries initiate this process by submitting the IPFS hash of the required documents. The function then checks if the tannery is registered and not already certified by utilizing the mappings of registered and certified tanneries. If the conditions are met, an event is triggered to inform the LWG certification organization of the available certification requests. This step is crucial for all slaughterhouses, tanneries, and manufacturers as it is a prerequisite for their participation in the production phase.

**Algorithm 3. Submitting certification request.**



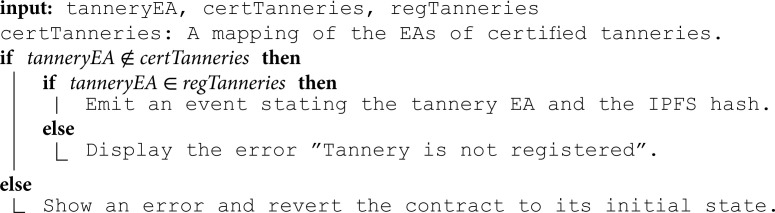



**Approving certification requests:** After a successful off-chain inspection process, Algorithm 4 is used to approve the LWG certification requests. This function is executed by the LWG organization, and it requires the address of the tannery and the validation period of the certification. The expiration timestamp is calculated based on the current blockchain time and stored on-chain. An event is then triggered to indicate the tannery address and the expiration time of the certification. The validation period is essential to guarantee that stakeholders consistently maintain compliance with sustainability standards over time, thereby enhancing the credibility of the certification system. This expiration value is later checked before any other function is executed to ensure that expired certifications cannot be misused. By periodically validating certifications and adding tannery addresses to the mapping of certified stakeholders, this algorithm contributes significantly to establishing a trusted proof of sustainability. It ensures that only tanneries meeting LWG certification standards are authorized to participate in the production phase, thereby enhancing transparency and trustworthiness in the supply chain.

**Algorithm 4. Approving certification request.**



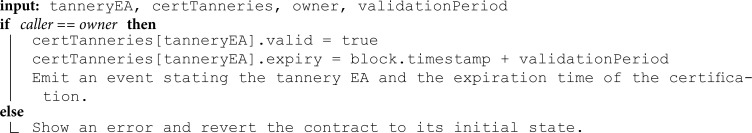



While the current design authorizes a single certifier to issue certifications, the system can be extended to support a multi-agency approval model, where multiple certification bodies must approve a request before it becomes valid. This enhancement would reduce reliance on any single authority and improve the integrity of the certification process. Furthermore, since all certification actions are recorded immutably on-chain, the system inherently supports public auditability and transparency of certifier decisions.

**Revoking certifications:** In cases where violations are detected during inspection visits or certifications are expired, certification organizations have the authority to revoke obtained certifications at any time. Algorithm 5 shows the function executed by the LWG certification organization for revoking certifications obtained by tanneries. This algorithm begins by verifying if the tannery holds a valid certification. If confirmed, it proceeds to remove the tannery record from the mapping of certified tanneries and triggers an event indicating the revocation.

This function is restricted to certification organizations. Restricting access to authorized entities ensures that certifications are revoked only under legitimate circumstances, thereby ensuring the credibility and reliability of the certification processes. Additionally, by enabling the revocation of certifications in response to detected violations, this algorithm contributes to maintaining the trustworthiness of sustainability certifications within the supply chain.

**Algorithm 5. Revoking certification.**



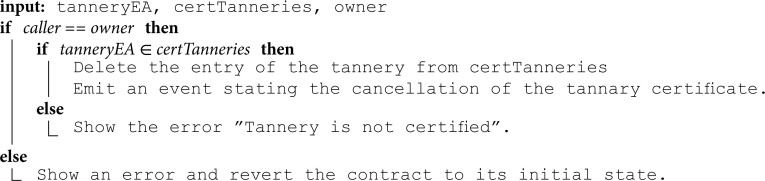



**Authorizing the smart contract to sell products:** Algorithm 6 represents the function executed by tanneries to authorize the smart contract to sell their leather lots. Registered and certified tanneries are authorized to initiate this function by specifying crucial details such as the address of the lot, its price, size, and the address of the associated hide lot used in production. Initially, the function validates the registration and certification status of the tannery. It also checks that the certification has not expired by comparing the current blockchain timestamp against the stored expiration timestamp. If the certification is no longer valid, the function is aborted and an error is returned. Subsequently, the function verifies the provided hide lot address by ensuring that the tannery address matches the buyer address of this hide lot. The buyer’s address is logged in the hide lots mapping during the execution of the purchasing function outlined in Algorithm 7. The size of the lots can vary; they can consist of either a single leather piece or multiple pieces with no restrictions. However, each lot will have just one address, meaning that the lot must be purchased in its entirety and cannot be divided. The size can also depend on a previous agreement between the buyer and the seller. If all conditions of the function are met, the leather lots mapping is updated with the entered inputs, and an event is emitted to notify relevant stakeholders. This process ensures that only certified and authenticated tanneries can register their leather lots for sale, thereby fostering transparency and trust within the supply chain.

**Algorithm 6. Authorizing the smart contract to sell products.**



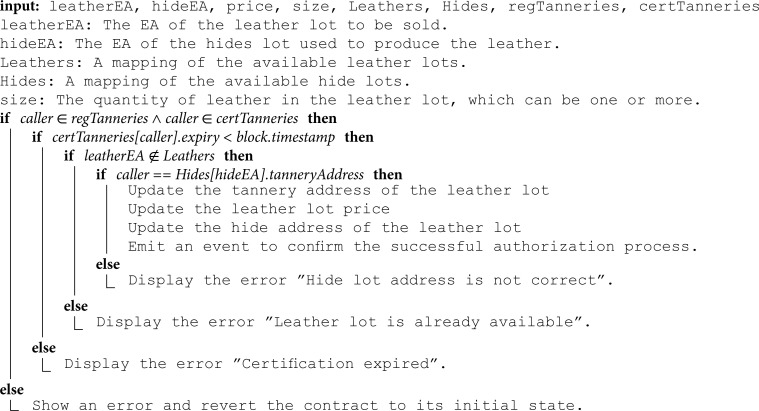



**Buying products:** Algorithm 7 describes the function that enables stakeholders and consumers to purchase products. This function can be executed by registered and certified manufacturers to buy leather from tanneries. Initially, the function verifies that the manufacturer holds a valid certification by checking that the current blockchain timestamp is within the stored certification validity period. If the certification has expired, the transaction is blocked and an error is returned. Next, the function verifies the availability of the leather lot by checking the leather mapping to ensure it is authorized for sale, not already sold, and produced by the specified tannery address. If these conditions are met and the provided Ether amount is enough, the manufacturer address and the availability status will be updated in the leather lot mapping. Additionally, the required Ether will be transferred to the tannery, and an event will be triggered to signify the transaction.

**Algorithm 7. Buying products.**



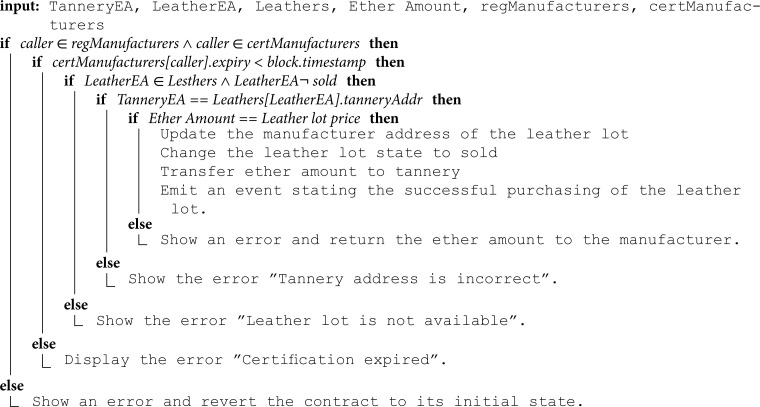



Through the utilization of logs, conditions, and mappings within the smart contracts, end consumers gain the ability to trace leather products back to their original materials as well as the stakeholders involved in their production. Each material type has a corresponding mapping that contains essential details such as the buyer, previous producer, and the previous material used in production. This enables end consumers to verify the accuracy of the shared information.

For instance, the buyer and previous stakeholder addresses are crucial for ensuring data accuracy. They are used by the functions to confirm that the previous material was indeed purchased by the buyer from the original seller. Additionally, consumers can utilize the mapping of the leather products to retrieve information about the purchased finished product and its manufacturer. The finished product address can then be used to access details about the leather lot used and the corresponding tannery. By consistently leveraging mappings in this manner, consumers can trace the products back to their initial raw materials and their source stakeholders.

Similarly, consumers can retrieve stakeholders’ addresses from the materials mappings and verify their certifications using the mappings of certified stakeholders. This approach enables consumers to validate all materials and stakeholders involved in the production processes. Consequently, this mechanism ensures the traceability of products from the farm to the consumer while providing trusted proof of sustainability throughout all production stages. The security and reliability of this process are guaranteed by the immutable nature of smart contracts and blockchain technology, where all data are immutably recorded and cannot be altered.

## 5 Testing and validation

After the development and implementation of the smart contracts, this section provides the details of the testing procedures conducted and presents the resulting outputs of the functions and events within our smart contracts. We test and validate the Registration, Certification, and Traceability smart contracts, deploying each one in the Remix IDE environment. The primary objective is to ensure the correct deployment and execution of functions within these smart contracts. Additionally, we verify that the output of each function confirms the expected behavior. Each smart contract has an owner, identified by the Ethereum address of the entity deploying the contract to the blockchain. Unauthorized function calls are prevented through the use of modifier functions for restricted operations. In the event of an unauthorized call, an error notification is triggered, and the smart contract is reverted to its original state. Our smart contracts code also incorporates events, which play a crucial role in maintaining data provenance and traceability. These events enable easy tracking of actions performed by stakeholders, with each stakeholder’s Ethereum address included in the event output. Table 1 lists the Ethereum address of the participating stakeholders interacting with the smart contracts, which serves as a reference during testing and validation. The subsequent sections provide a detailed execution of the functions and their corresponding outputs.

**Table 1:** The Ethereum address of each stakeholder used in the testing scenario.

Stakeholder	Ethereum Address
Farm	0x4B20993Bc481177ec7E8f571ceCaE8A9e22C02db
Slaughterhouse	0x78731D3Ca6b7E34aC0F824c42a7cC18A495cabaB
Tannery	0x617F2E2fD72FD9D5503197092aC168c91465E7f2
Manufacturer	0x17F6AD8Ef982297579C203069C1DbfFE4348c372
Retailer	0x5c6B0f7Bf3E7ce046039Bd8FABdfD3f9F5021678

### 5.1 Submitting the registration request

**Fig 6:**
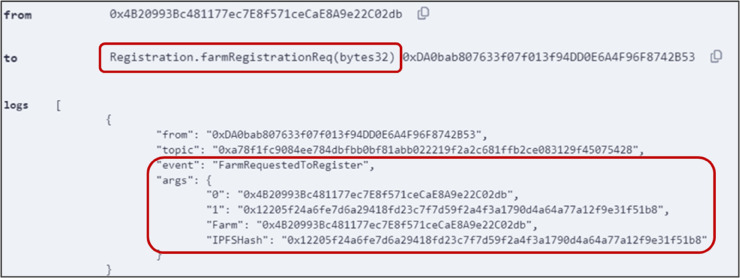
Output logs showing successful execution of *farmRegistrationReq()* function.

The process begins with the registration of stakeholders using Algorithm 1. The farm executed the *farmRegistrationReq()* function by providing the IPFS hash. Fig 6 shows the successful execution of the function, along with corresponding logs and events. Subsequently, similar functions have been executed successfully to register all other stakeholders.

### 5.2 Approving the registration request

**Fig 7:**
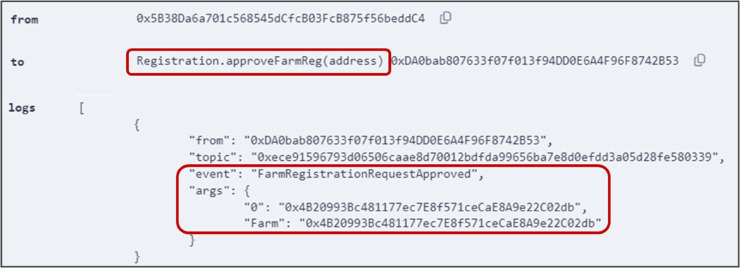
Output logs showing successful execution of *approveFarmReg()* function.

Fig 7 shows a successful execution of *approveFarmReg()* function described in Algorithm 2. This function was executed by the government registrar to officially register the farm on the system. Unauthorized attempts to execute the function from other callers will display an error.

### 5.3 Submitting certification request

**Fig 8:**
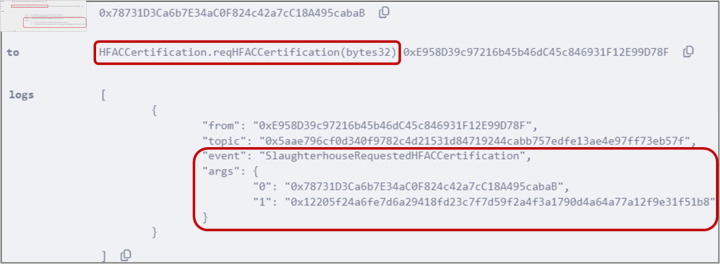
Output logs showing successful execution of *reqHFACCertification()* function by the slaughterhouse.

Stakeholders requiring certifications must submit certification requests, as outlined in Algorithm 3. To request HFAC certification, the slaughterhouse executed the *reqHFACCertification()* function by providing the IPFS hash value as an input. Fig 8 shows the event triggered after the successful execution of the function where the slaughterhouse address and the IPFS hash are displayed. Similarly, the *reqLWGCertification()* and *reqOEKOTEXCertification()* have been executed to certify the tannery and the manufacturer.

### 5.4 Approving certification request

**Fig 9:**
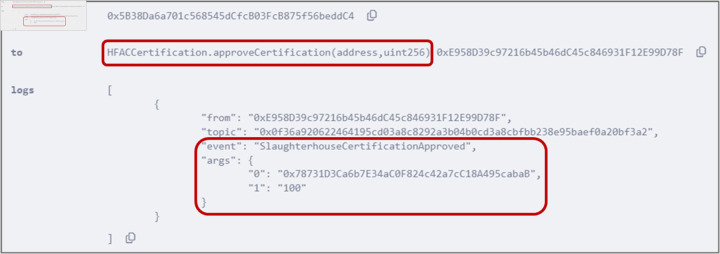
Event emitted by *approveCertification()* function, displaying the slaughterhouse address and certificate validation period.

The HFAC organization executes the *approveCertification()* function described in Algorithm 4 to approve the certification requested by the slaughterhouse. Parameters including the address of the slaughterhouse and the validation period of the certification are passed as inputs to the function. Fig 9 shows the event triggered, which indicates the successful certification of the slaughterhouse along with the certification validation period.

### 5.5 Revoking the certification

**Fig 10:**
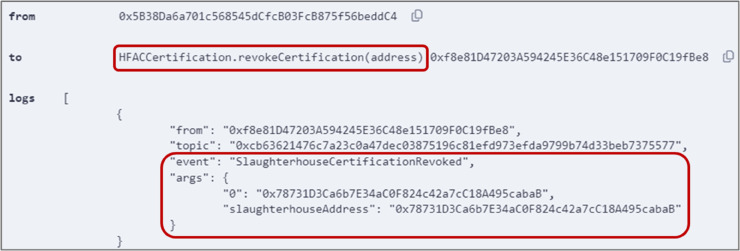
Output logs showing successful execution of *revokeCertification()* function.

Certification agencies can revoke obtained certifications if necessary at any time through the execution of the *revokeCertification()* function outlined in Algorithm 5. Fig 10 shows the successful execution of the function by the certification agency to revoke the HFAC certification obtained by the slaughterhouse. The address of the slaughterhouse was passed as an input, and an event was triggered confirming that the slaughterhouse certification was revoked.

### 5.6 Authorizing the smart contract to sell leather products

**Fig 11:**
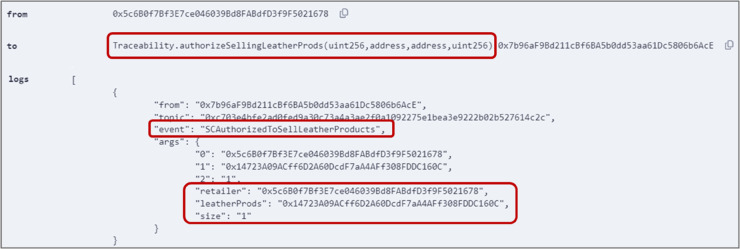
Output logs of *authorizeSellingLeatherProds()* function, displaying the address and price of the product.

**Fig 12:**
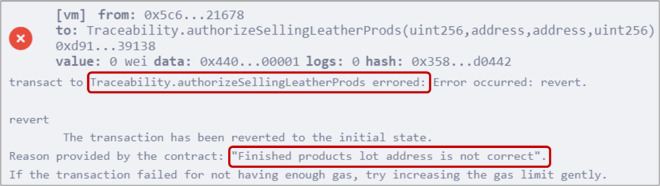
Error message indicating incorrect entry of the previous material address.

Stakeholders can sell their available products by authorizing the smart contract to do that, as outlined in Algorithm 6. For instance, the retailer executed the *authorizeSellingLeatherProds()* function by providing the size and price of the available leather products lot, its Ethereum address, and the address of the finished product lot from the manufacturer. The function triggered an event containing all relevant information, as shown in Fig 11. Additionally, any errors in the provided information, such as false details related to the previous product bought from the manufacturer, will be displayed, as shown in Fig 12, ensuring authenticity and traceability.

### 5.7 Purchasing leather products by the end consumer

**Fig 13:**
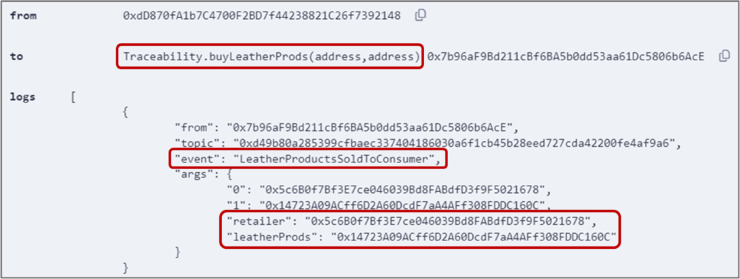
Output logs of *buyLeatherProds()* function, showing successful purchase of leather product.

**Fig 14:**
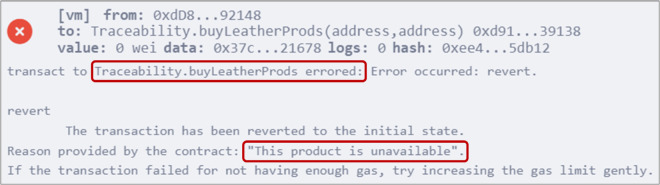
Error message displayed when the end consumer attempts to purchase unavailable products.

The *buyLeatherProds()* function, detailed in Algorithm 7, allows any consumer to purchase leather products from a retailer. To make a purchase, the consumer executed the function by providing the addresses of the retailer and the desired product as inputs. Fig 13 displays the output event confirming the successful purchase of the product along with the retailer and the product addresses. In cases of insufficient Ether amount or product unavailability, error messages will be displayed, as illustrated in Fig 14.

### 5.8 Ensuring trusted traceability and proof of sustainability

**Fig 15:**
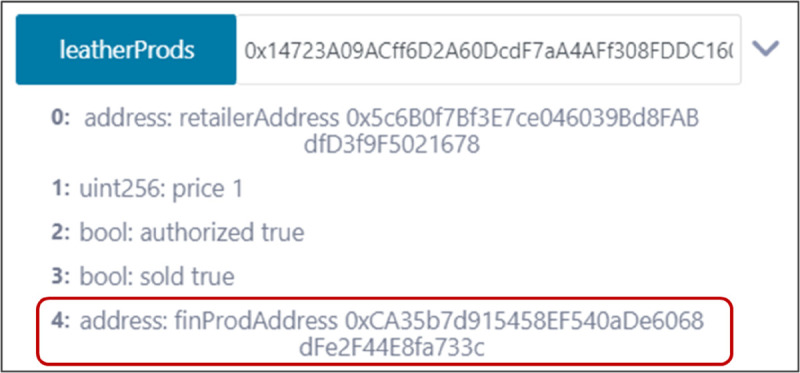
Output of the leather products mapping displaying traceability information for a leather product.

The outputs presented in the preceding subsections offer an overview of how our system ensures trusted traceability and sustainability across the leather supply chain through events triggered and logs saved on the ledger. By utilizing mappings for each type of material, crucial details such as the producer, buyer, and previous material source are recorded and verified at each step. For example, Fig 15 shows the output of the leather products mapping for a leather product purchased by an end consumer. This demonstrates how the product path can be traced back to its original raw material source. By utilizing the finished product address and its associated mapping, the traceability process extends until reaching the initial source.

**Fig 16:**
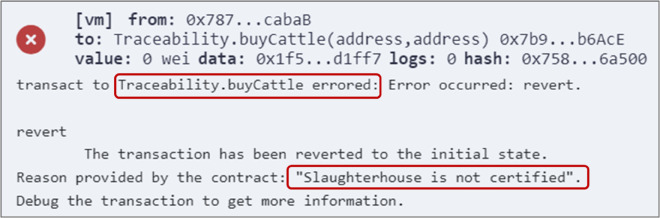
Error message displayed when a slaughterhouse attempts to buy cattle without being certified.

**Fig 17:**
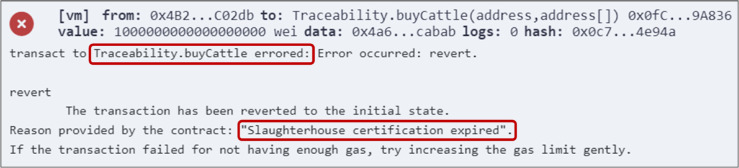
Error message displayed when a slaughterhouse with an expired certification attempts to buy cattle.

Additionally, proof of sustainability is accomplished by forcing stakeholders to obtain the relevant sustainability certifications to be authorized to participate in the production and trading processes. These certifications have a validation period and can be revoked at any time if any violations are detected during periodic or unannounced inspection visits. Moreover, consumers can utilize the mappings of certified stakeholders to verify their certification status and validation period, further enhancing transparency. Fig 16 illustrates a sample error message displayed when a slaughterhouse attempts to buy cattle or sell hides without being certified. This ensures the commitment to environmentally responsible practices throughout the entire production process. In addition to verifying whether a stakeholder is certified, the system also verifies certification validity and restricts access once a certification has expired. Stakeholders with expired certifications are automatically restricted from performing any actions. Fig 17 shows the error displayed when a stakeholder with an expired certification attempts to buy a material. This error confirms that the system prevents participation beyond the allowed validity period, further ensuring sustainability compliance across the supply chain.

The trustworthiness of our system is ensured by the immutable nature of blockchain technology, which ensures that all data and logs are recorded on the ledger in a way that cannot be altered. With tamper-proof integrity and robust traceability mechanisms, our system provides a reliable record of every transaction and interaction within the supply chain. Moreover, all data transactions are secured using digital signatures, further enhancing the system integrity and ensuring the accountability and authenticity of information.

## 6 Discussion and analysis

Following the successful testing of our solution, this section provides a discussion and evaluation of our blockchain-based solution. We provide cost and security analyses to assess its reliability and feasibility for use in real-world scenarios. Furthermore, we compare our approach with the existing solutions, and we discuss how our solution can be extended and generalized to other systems.

### 6.1 Cost analysis

This subsection presents the cost analysis of our Ethereum smart contracts code and function calls. On the Ethereum blockchain, each transaction requires a gas fee, which is paid in Ether. Remix IDE proves invaluable for estimating gas costs due to its user-friendly interface. The total cost of each function comprises of two components: transaction and execution gas costs. Execution costs involve the actual execution of function code, including storage and state manipulation within the smart contract [30]. On the other hand, transaction costs cover factors such as contract deployment and data transmission to the blockchain network [30]. Additionally, the gas price denotes the Ether cost per unit of gas, measured in Gwei. Gas prices increase during network congestion as miners prioritize transactions based on gas prices [31]. Consequently, users opting for higher gas prices experience expedited transaction processing.

Tables 2, 3, and 4 display the gas costs associated with various functions utilized in the smart contracts, along with their conversion into fiat currency (USD) using different blockchain platforms. The gas prices are subject to fluctuation over time, and for this analysis, an average gas price of 17 Gwei was utilized based on pricing data accessed on April 01, 2024 [32].

**Table 2:** Execution costs of the Registration smart contract functions.

Function Name	Transaction Gas	Transaction Fee (Ether)	Cost Using Ethereum (USD)	Cost Using Cardano (USD)	Cost Using zkSync (USD)
*regisrtationReq()*	25869	0.00044	1.595	0.00029	0.00003
*approveReg()*	45530	0.00077	2.792	0.00050	0.00004
*denyReg()*	23604	0.00040	1.450	0.00026	0.00002

**Table 3:** Execution costs of the Certification smart contracts functions.

Function Name	Transaction Gas	Transaction Fee (Ether)	Cost Using Ethereum (USD)	Cost Using Cardano (USD)	Cost Using zkSync (USD)
*requestCertification()*	31977	0.00054	1.958	0.00035	0.00003
*approveCertification()*	46135	0.00078	2.828	0.00051	0.00004
*denyCertification()*	23205	0.00039	1.414	0.00025	0.00002
*revokeCertification()*	23736	0.00040	1.450	0.00026	0.00002

**Table 4:** Execution costs of the Traceability smart contract functions.

Function Name	Transaction Gas	Transaction Fee (Ether)	Cost Using Ethereum (USD)	Cost Using Cardano (USD)	Cost Using zkSync (USD)
*authorizeSellingCattle()*	97831	0.00166	6.019	0.00108	0.00010
*buyCattle()*	57847	0.00098	3.553	0.00064	0.00006
*authorizeSellingHides()*	107388	0.00183	6.636	0.00119	0.00011
*buyHides()*	79763	0.00136	4.931	0.00088	0.00007
*authorizeSellingLeatherProds()*	101037	0.00172	6.237	0.00111	0.00010
*buyLeatherProds()*	44524	0.00076	2.756	0.00049	0.00004

Overall, Ether and gas costs are generally low for all functions, especially those in the registration and certification smart contracts, as they primarily trigger events without altering the state of variables. Conversely, functions within the traceability smart contract tend to result in higher transaction gas, particularly the authorization functions. This is because these functions update numerous variables and mappings within the smart contracts in order to achieve materials tracking throughout the supply chain.

When considering the costs in USD, they are notably high for most functions and cannot be negligible. This is primarily due to the current high cost of Ether, where 1 Ether equals 3,625 USD. The rising cost of Ether and its volatility presents a significant challenge in the Ethereum network, which leads developers to seek alternative solutions for designing and implementing their solutions. One such solution is implementing the smart contracts on alternative blockchains such as zkSync and Cardano. zkSync is a trustworthy, secure, and user-centric Layer 2 blockchain that is built on top of Ethereum mainnet. It works by processing transactions off-chain and then periodically settling them back on the main blockchain [33]. This approach minimize network congestion and lower gas fees by allowing a large volume of transactions to be handled off-chain. zkSync promises to drastically reduce transaction costs on the Ethereum blockchain to a negligible fraction, as the current price is set at just $0.0574. This is clearly illustrated in the tables, where all costs remain below $0.0001. Another option is using the Cardano platform, which is a decentralized blockchain built on a proof-of-stake consensus mechanism that eliminates the need for resource-intensive mining processes [34]. It offers a reliable and secure platform known for its focus on scalability, energy efficiency, and user experience. By deploying the smart contracts on the Cardano platform, users can expect significant reductions in transaction costs compared to other blockchain networks, with the current price set at just $0.65. As demonstrated in the tables, transaction costs on Cardano are consistently lower than Ethereum, all falling below $0.002. This underscores the cost-efficiency and accessibility of utilizing Cardano and zkSync for various blockchain operations. Another viable solution to address transaction fees is the utilization of a private blockchain network. In private blockchain networks, the gas price that miners accept can be configured to zero, where nodes can freely engage with smart contracts without incurring any transaction fees [33]. Our solution is designed to be compatible with any Ethereum Virtual Machine (EVM)-based blockchain. Therefore, it can be easily deployed on private Ethereum blockchains and Layer 2 solutions, which provide users with flexibility and cost-efficient solutions for their blockchain operations.

In addition to deploying smart contracts on low-cost blockchain platforms, batch processing represents another promising cost optimization strategy. Instead of executing repetitive transactions individually, batch processing allows multiple operations, such as authorizing or purchasing product lots, to be grouped into a single transaction using array-based inputs. This significantly reduces the overhead gas consumed per operation. As shown in Table 5, batching led to gas reductions of up to 26.74% for *authorizeSellingCattle()* and up to 68.68% for *buyCattle()* when processing 10 operations at once. The results also show that the percentage of gas saved increases as more items are processed together, making batch processing especially valuable in high-throughput environments where gas fees accumulate rapidly. Unlike platform migration, batch optimization requires only minimal changes to the smart contract logic. This makes it a practical and flexible solution for improving cost-efficiency without compromising decentralization or transparency.

**Table 5:** Comparison of gas usage with and without batch processing for *authorizeSellingCattle()* and *buyCattle()* functions.

Batch Size	*authorizeSellingCattle()*			*buyCattle()*		
	Gas Usage (Without Batch)	Gas Usage (With Batch)	Reduction	Gas Usage (Without Batch)	Gas Usage (With Batch)	Reduction
2	195,304	167,997	13.9%	115,358	73,947	35.9%
5	488,260	373,317	23.5%	288,395	113,958	60.5%
10	976,520	715,518	26.7%	576,790	180,644	68.7%

### 6.2 Security analysis

**Fig 18:**
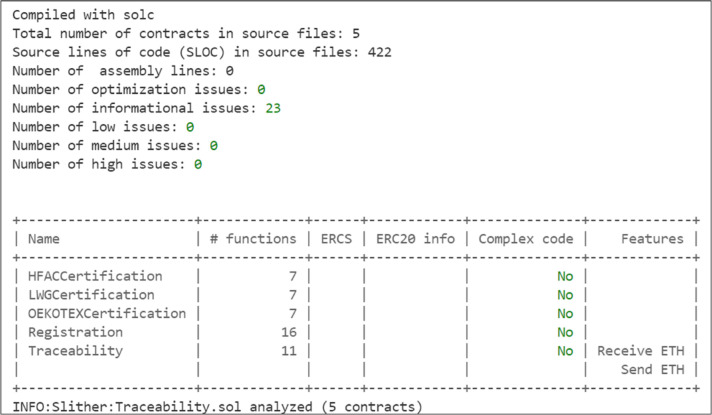
Output of Slither tool showing vulnerability-free smart contracts.

Blockchain networks rely on cryptographic principles to provide high levels of security, resilience, and robustness. However, smart contracts can be vulnerable to exploitation. It is essential to check smart contracts for software bugs that could make them highly vulnerable. To address this, we utilized the Slither [35] analysis tool, which is a Python-based static analysis framework that detects vulnerabilities in Solidity smart contracts, offers visual contract details, and enables developers to quickly find and fix vulnerabilities. Our smart contract codes underwent thorough testing using the Slither tool. After multiple iterations of modification, addressing identified vulnerabilities, the smart contracts were confirmed to be free of all vulnerabilities except for few informational issues, as shown in Fig 18. This gives us confidence in the resilience of our smart contracts against known bugs and vulnerabilities.

On the other hand, blockchain technology offers inherent security features that make it an ideal solution for various applications. By integrating blockchain, developers can create trusted, secure, and tamper-resistant systems. One of the key advantages of blockchain is its ability to ensure data integrity and consistency throughout the chain. In the context of leather products, traceability and integrity are maintained through an event-based approach. Each transaction, from raw material production to product purchase, is meticulously recorded and stored on an immutable ledger. The immutability of blockchain, achieved through sequential hashing, guarantees that once information is added to the chain, it remains unalterable, further enhancing reliability. Moreover, Ethereum smart contracts improve accountability by utilizing features such as modifiers. These modifiers empower specific actors to execute designated functions. Additionally, the decentralized nature of the blockchain ensures non-repudiation, which prevents actors from denying their actions on the chain. It also mitigates the risk of denial-of-service attacks, which ensures information availability to all stakeholders. Overall, blockchain technology ensures a secure and accountable framework for various applications, which in turn promotes trust, reliability, and integrity.

To further support transparency and reduce trust dependence on individual certification agencies, all certification-related actions, including requests, approvals, denials, and revocations, are immutably logged on-chain. These records are publicly accessible and verifiable, which enables stakeholders to audit the behavior of certifiers at any time. This public auditability acts as a natural deterrent against potential misconduct and provides a foundation for future extensions such as multi-agency approvals or decentralized governance models. As a result, the system fosters accountability and strengthens stakeholder trust, even in scenarios where a single certifier is responsible for issuing certifications.

### 6.3 Comparison with existing solutions

Several research studies across various sectors have explored and proposed solutions to enhance supply chain traceability and sustainability. Table 6 presents a comparative analysis between our proposed solution and other existing approaches in different sectors.

**Table 6:** Comparison of our proposed system with existing solutions in the literature.

	Targeted Industry	Blockchain Role	Smart Contract Integration	Traceability Scope	Certification Handling	Sustainability Verification	Implementation & Testing
[25]	Fashion	Records lifecycle data and environmental impact on blockchain	NA	Full lifecycle	Not integrated	LCA-based, no enforcement	Theoretical framework
[26]	Textile	Provenance + production steps recorded on blockchain	Yes	Production & transformation steps only	Not integrated	Not supported	Full implementation + testing
[27]	Luxury brands	Adds anti-counterfeit data + carbon claims	NA	Full lifecycle + anti-counterfeit labels	Not integrated	Lifecycle tracking, no on-chain verification	Theoretical framework
[36]	Halal food	Links blockchain with halal certification schemes	NA	Full lifecycle	Indirect (policy-focused)	Not supported	Theoretical framework
[37]	Food	Consortium blockchain for data integrity + public chain for transparency + oracle layer for external data integration	Yes	Full lifecycle via sensor inputs	Not integrated	IoT-based, no certification agency	Theoretical framework
**Our Solution**	Leather	Enforces traceability and sustainability via smart contracts, immutable logs, and on-chain certification controls	Yes	Full lifecycle (farm to consumer)	Enforced via certification + expiry + revocation	Required for participation, verified on-chain by certification agencies	Full implementation + testing and evaluation

The solutions proposed by Shou et al. [25] and Al-Issa et al. [27] illustrate how a blockchain-based traceability solution can enhance the sustainability of products. They explained the tracking mechanisms that demonstrate how products can be monitored throughout their entire lifecycle. Through the data stored on the blockchain, stakeholders gain access to extensive information on practices and materials, enabling sustainability assessment. However, these solutions lack a method to verify the accuracy of recorded details, thereby failing to prove sustainability claims. Additionally, they offer theoretical insights without practical implementation or testing. Similarly, Bux et al. [36] examined the barriers and opportunities associated with using certification and blockchain tools to improve the reliability and traceability of Halal food products. However, their study focused on how certification usage could contribute to establishing a trusted and traceable system without technical or implementation details.

While Perez et al. [26] offered detailed implementation and testing cases, their focus was solely on product traceability without considering sustainability aspects. Furthermore, Cao et al. [37] introduced a multi-layered blockchain-based architecture aimed at improving sustainability throughout the food supply chain. They integrated an oracle layer to facilitate connectivity between the blockchain and external systems, including IoT devices, for collecting sustainability-related data about products and their situations. However, they are unable to fully confirm the sustainability of products at all stages, as the sustainability-related data remains stored on the chain without validation or oversight that can prevent stakeholders’ participation in case of detected violations.

Overall, while existing solutions address some of the challenges related to traceability and sustainability in various industries, there remains a significant gap that underscores the need for our proposed solution. By incorporating sustainability certification agencies, our solution offers trusted proof of sustainability to ensure that all participating stakeholders are monitored and certified for their sustainable and ethical practices at every stage of production. Moreover, existing studies have predominantly been theoretical without practical implementation and testing. This highlights the need for a solution that offers a practical implementation and testing details, along with thorough cost and security analysis to evaluate its reliability and feasibility.

### 6.4 Generalization

The blockchain-based solution tailored for the leather supply chain presented in this study not only addresses the critical need for trusted traceability and sustainability within this specific industry but also offers a scalable model applicable to various other sectors. While our focus has been on the leather sector, the fundamental architecture of our solution, including the integration of Ethereum smart contracts and certification agencies, can be readily applied to other sectors where ensuring ethical and sustainable practices is paramount. For instance, industries such as cosmetics, electronics, agriculture, and pharmaceuticals can benefit from similar solutions to track the provenance of raw materials, ensure ethical sourcing practices, and verify product authenticity. In the cosmetics industry, our solution can facilitate the tracking of ingredients sourced from sustainable suppliers, and verified through certifications such as Fair Trade or Cruelty-Free.

Similarly, in the agriculture sector, our solution can be utilized to track the journey of food products from farm to market, which ensures food safety and authenticity. For instance, it can enable farmers to record details about the cultivation process, including the use of pesticides and fertilizers, which can be verified through certifications such as Organic or Non-GMO. The key difference lies in the adaptation of smart contracts to accommodate the verification and validation processes specific to the certification requirements of each industry. While the core functionality remains consistent across applications, such as recording transactions on the blockchain and automating supply chain interactions, the smart contracts would need to be tailored to handle the nuances of different certification standards and verification protocols. The only differences for the smart contracts will be in the specific rules generated for each participant and the details of the product that needs to be stored. Otherwise, the same algorithms and logic can be applied.

Additionally, even for the leather industry in our solution, it is not limited to the three mentioned certifications but can be easily adapted for use with other certification agencies or additional ones. By leveraging blockchain technology and smart contracts, our solution offers a versatile platform for ensuring traceability and sustainability across diverse industries while accommodating the intricacies of certification processes unique to each sector.

### 6.5 Challenges and limitations

Blockchain technology, while offering numerous advantages, also presents several challenges and limitations that must be addressed for widespread adoption and effectiveness. One critical limitation is the unupgradable feature of the smart contracts which poses a significant challenge. It prohibits any modifications or updates, even to fix bugs or incorporate new features. This limitation necessitates thorough initial planning and testing to ensure the smart contracts meet the long-term requirements of the solution. Another challenge is the immutability of blockchains, where once information is recorded, it cannot be altered or removed. While this feature ensures data integrity, it presents challenges in correcting inaccuracies or errors made during data entry, potentially impacting the supply chain. In addition, the current work lacks real-world deployment and direct engagement with leather industry stakeholders. While the system has been technically validated through simulation and testing, practical evaluation in operational environments is still essential to confirm its effectiveness and usability. As part of future work, we plan to conduct real-world deployments with selected leather manufacturers and certification bodies to gather user feedback, assess usability, and evaluate the system’s performance in real supply chain settings.

Moreover, our blockchain-based solution faces challenges related to its practical implementation and scalability in the global supply chain. Ethereum proof-of-work consensus mechanism requires individual nodes to process every transaction, which could lead to potential congestion and limited throughput, particularly in large-scale manufacturing scenarios where multiple suppliers and stakeholders across different regions are involved. This results in slow transaction processing and high costs during peak network usage. The transition to Ethereum 2.0 proof-of-stake model and the introduction of the sharding mechanism effectively address the scalability challenges of the Ethereum network. The proof-of-stake consensus mechanism allows faster transaction finality and higher throughput by reducing the computational overhead required for transaction validation [38]. Sharding breaks down the blockchain into smaller pieces to enable parallel processing of transactions across multiple nodes, thereby increasing the network overall scalability and capacity [39]. These advancements in the Ethereum 2.0 model ensure the feasibility of our blockchain-based solution for practical implementation in real-world scenarios.

The rising cost of Ether in the Ethereum network presents another challenge to the accessibility and affordability of blockchain solutions. These costs are high due to the current high cost of Ether, and they increase with transaction volume and network congestion which hinder adoption in high-transaction environments. Addressing these cost issues is essential for the practical deployment of blockchain solutions in global supply chains. While Ethereum 2.0 proof-of-stake mechanism reduces transaction costs by eliminating the need for computationally intensive mining processes, Layer 2 solutions further provide effective approaches to handle high volumes of transactions with lower costs and faster speeds. By offloading and processing many transactions outside the main blockchain, layer 2 solutions such as zkSync and Polygon can significantly reduce congestion and lower costs. For instance, the current price of Polygon in USD is $0.64, which can significantly reduce total transaction costs compared to Ether. Our smart contracts can be easily deployed on these platforms as they are compatible with any EVM-based blockchain.

Lastly, another limitation of the current design is its reliance on the integrity of individual certification agencies. While all certification actions are publicly logged on-chain to enhance transparency, the certification process itself is currently controlled by a single authority. In future versions, the system can be extended to incorporate multi-agency verification or decentralized governance models, where certifications require approvals from multiple independent entities to mitigate the risk of certifier bias or misconduct. The system also inherits the oracle problem, as it relies on external certifiers to honestly report real-world inspection outcomes. This highlights the need for secure, verifiable bridges between physical events and on-chain data in future implementations.

## 7 Conclusion

In this paper, we have proposed a blockchain-based solution tailored to address the traceability and transparency challenges within the leather products supply chain, while also addressing environmental concerns by ensuring sustainability compliance and ethical practices. Our solution offers proof of sustainability across every stage of the leather supply chain by providing certification, thus establishing transparency and fostering accountability among stakeholders. We have developed five Ethereum smart contracts leveraging the immutability and transparency of the blockchain network to automate the registration, certification, and materials exchange processes. These smart contracts use modifiers, mappings, and events to facilitate efficient tracing and tracking of the supply chain at every stage. We tested the functionality of the system and illustrated how end consumers can trace back any leather product to its origin materials while proving sustainable and ethical practices throughout the production stages. Furthermore, our cost analysis revealed that implementing the solution using Ethereum while utilizing the zkSync Ethereum network, resulted in a maximum cost of $0.0001. Additionally, our security analysis, conducted using the Slither tool, confirmed the resilience of our solution to known security attacks and its fulfillment of cybersecurity requirements. Lastly, we compared our solution with existing solutions, and we identified a gap that our solution effectively addressed. Additionally, we discussed how our solution can be generalized and extended to other systems experiencing similar issues or requiring similar specifications. As a future work, we plan to develop and build an end-to-end DApp and deploy it on the zkSync mainnet.
